# Valorization of *Juglans regia* Leaves as Cosmeceutical Ingredients: Bioactivity Evaluation and Final Formulation Development

**DOI:** 10.3390/antiox11040677

**Published:** 2022-03-30

**Authors:** Nermine Besrour, Taofiq Oludemi, Filipa Mandim, Carla Pereira, Maria Inês Dias, Marina Soković, Dejan Stojković, Olga Ferreira, Isabel C. F. R. Ferreira, Lillian Barros

**Affiliations:** 1 Centro de Investigação de Montanha (CIMO), Instituto Politécnico de Bragança, Campus de Santa Apolónia, 5300-253 Bragança, Portugal; besrourn@gmail.com (N.B.); taofiq.oludemi@ipb.pt (T.O.); filipamandim@ipb.pt (F.M.); carlap@ipb.pt (C.P.); maria.ines@ipb.pt (M.I.D.); iferreira@ipb.pt (I.C.F.R.F.); 2 Nutrition and Bromatology Group, Department of Analytical Chemistry and Food Science, Faculty of Science, Univeridade de Vigo, 36310 Ourense, Spain; 3 Department of Plant Physiology, Institute for Biological Research Siniša Stanković—National Institute of Republic of Serbia, University of Belgrade, Bulevar Despota Stefana 142, 11000 Belgrade, Serbia; mris@ibiss.bg.ac.rs (M.S.); dejanbio@ibiss.bg.ac.rs (D.S.)

**Keywords:** *Juglans regia*, phenolic compounds, cosmeceuticals, bioactivity

## Abstract

The cosmetic industry is constantly searching for bioactive ingredients, namely, those obtained from natural sources with environmentally friendly connotations and less toxic effects. A previous study of our research group optimized the extraction of phenolic compounds from *Juglans regia* by heat-assisted extraction. Due to its richness in different phenolic compounds, the present work aimed to develop a formulation containing *J. regia* leaf extract. The extract’s antioxidant, anti-tyrosinase, antimicrobial, anti-inflammatory, wound healing, cytotoxicity, and photostability properties were evaluated. The extract was then incorporated into an O/W base cream, followed by characterization of the final formulation in terms of its antioxidant properties, phenolic composition, and stability over time and at different storage conditions. The most abundant compounds in the hydroethanolic extract were 3-*O*-caffeoylquinic acid (18.30 ± 0.04 mg/g), quercetin-*O*-pentoside (9.64 ± 0.06 mg/g), and quercetin 3-*O*-glucoside (6.70 ± 0.19 mg/g). Besides those, the extract presented antioxidant, anti-inflammatory, wound closure, and antibacterial effects against several skin pathogens. In addition, HaCaT cell viability was maintained up to 98% at 400 µg/mL. Within *Proteus vulgaris*-infected HaCaT cells, the extract also presented an over 40% bacterial mortality rate at its nontoxic concentration (200 µg/mL). After incorporating the extract, the obtained formulation presented a good physicochemical profile over time and at different storage conditions while also maintaining its antioxidant effect; as such, it can be considered stable for topical application. Future work to evaluate its performance in terms of skin permeation and detailed toxicological studies with a focus on regulatory requirements, involving skin irritation, eye irritation, genotoxicity, photo-irritation, and dermal absorption, should be conducted, as the prepared formulation demonstrated relevant properties that deserve to be further explored.

## 1. Introduction

Cosmeceuticals are topical formulations in the form of oils, creams, and ointments containing biologically active ingredients with valuable medical drug-like benefits on the skin. The term “cosmeceutical” is still not legally recognized according to the Federal Food, Drug, and Cosmetic Act (FD&C Act) due to widespread misunderstandings regarding its definition and scope [1]. However, these products have unquestionably taken over the global personal care business. This significant growth in the worldwide cosmetic market is associated with their multifunctional benefits: antioxidant, UV-protective, anti-inflammatory, and antiaging activities [2,3]. This tendency is also reinforced by the increased consumer awareness regarding the environmental impact of cosmetic additives. It has shifted the preference towards sustainably sourced biomolecules from different natural matrices to develop innovative and efficient formulation ingredients that are safe, effective, and ecologically acceptable, compared to synthetic counterparts [4]. Several bioactive molecules, especially those belonging to the hydroxycinnamic acids, flavanols, anthocyanidins, and other phenolic compound groups, have demonstrated strong multifunctional bioactive properties [5,6,7]. These ingredients, if sustainably sourced, can provide a broad spectrum of functionality and become viable alternatives to the synthetic ingredients used in formulation development. 

Walnut (*Juglans regia* L.) is a widely distributed deciduous tree species found mainly in temperate zones and is popularly referred to as Persian walnut, white walnut, English walnut, or common walnut [8]. Extracts obtained from the different plant parts have presented multifunctional biological activities, namely, anti-inflammatory, antitumor, antiviral, and antimicrobial properties [9,10]. More interestingly, bioactive compounds such as alkaloids, phenolic acids, flavonoids, naphthalene derivatives, and carotenoids have been identified in this species and are responsible for the above-mentioned bioactive properties [11,12,13]. The use of inexpensive by-products such as shells, kernels, barks, green walnut husks (epicarp), and leaves arising from *J. regia* cultivation and processing as sources of structurally diverse extractable bioactive molecules has attracted much attention in recent years [14,15,16]. Due to the multifunctional bioactive properties of extracts obtained from *J. regia* by-products, there is an increased interest in commercializing these extracts as cosmeceutical additives. An increasing amount of scientific evidence supports the beneficial “cosmeceutical” effects of extracts obtained from the different parts of *J. regia* plants [17,18,19]. However, studies on formulation development using *J. regia* extracts are significantly lacking, as reviewed by Ribeiro et al. [20]. Hence, the present work aimed to chemically characterize *J. regia* leaf extract, obtained using a heat-assisted extraction method already optimized [21]. Several bioactive properties were evaluated: antioxidant, anti-inflammatory, anti-tyrosinase, and antimicrobial activity against skin pathogens. In addition, cytotoxicity in skin cell lines, photostability, and microbial invasion assays were assessed for the first time on the studied extract. Furthermore, the obtained extracts were incorporated in a base cream, and the formulation was stored at different temperature conditions (5 °C, 20 °C, and 40 °C). Then, different parameters of the formulation were evaluated over time, namely, pH, color, antioxidant activity, and the phenolic compound content quantified by HPLC. 

## 2. Materials and Methods

### 2.1. Standards and Reagents

Acetonitrile 99.9% was of high-performance liquid chromatography (HPLC) grade, supplied by Lab-Scan (Lisbon, Portugal), and methanol was of analytical grade from Pronalab (Lisbon, Portugal). 2,2-Diphenyl-1-picrylhydrazyl (DPPH) was obtained from Alfa Aesar (Ward Hill, MA, USA). Dulbecco’s modified Eagle’s minimum essential medium (DMEM), fetal bovine serum (FBS), penicillin, streptomycin, Griess reagent system (Promega), dimethyl sulfoxide (DMSO), sulforhodamine B (SRB), lipopolysaccharide (LPS), 3,4-dihydroxy-L-phenylalanine (L-DOPA), dexamethasone, and mushroom tyrosinase enzyme were obtained from Sigma-Aldrich Co. (Saint Louis, MO, USA). Muller Hinton broth (MHB) and Tryptic Soy Broth (TSB) medium were purchased from Biomerieux (Marcy l’Etoile, France). Blood agar with 7% sheep blood and Mac Conkey agar plates were purchased from Biomerieux (Marcy l’Etoile, France). The *p*-iodonitrotetrazolium chloride (INT) dye and phenolic compound standards were purchased from Sigma-Aldrich (St. Louis, MO, USA). 

### 2.2. Plant Material and Extraction Process

The *J. regia* dried leaves were purchased from Soria Natural, S.A., Soria, Spain. The samples were milled (60 to 20 mesh) and stored in a desiccator protected from light for subsequent analysis. The heat-assisted extractions (HAEs) were performed using a heating instrument with magnetic stirring in a thermostated water bath. The powdered samples (0.6 g) were extracted with 20 mL of 50.4% ethanol for 112.5 min at 61.3 °C. The above conditions were optimized [21]. Afterwards, the mixtures were filtered, followed by the evaporation of the solvent under reduced pressure (rotary evaporator Büchi R-210, Flawil, Switzerland) to obtain the dried extracts. 

### 2.3. Quantification of the Main Phenolic Compounds in the Extracts

The obtained extracts were re-dissolved in 20% aqueous ethanol at 10 mg/mL, filtered through a 0.22 µm nylon syringe filter, and analyzed by HPLC-DAD-ESI/MSn in a Dionex Ultimate 3000 UPLC system (Thermo Scientific, San Jose, CA, USA). The equipment consisted of a diode array detector coupled to an electrospray ionization mass detector, a quaternary pump, an auto-sampler (kept at 5 °C), a degasser, and an automated thermostated column section (kept at 35 °C). A Waters Spherisorb S3 ODS-2 C18 (3 µm, 4.6 mm × 150 mm, Waters, Milford, MA, USA) column was used. Data were collected simultaneously with DAD and in negative mode detection on a Linear Ion Trap LTQXL mass spectrometer (Thermo Scientific, San Jose, CA, USA), following a previously described procedure [22]. An Xcalibur^®^ data system (Thermo Scientific, San Jose, CA, USA) was used in data acquisition. Phenolic compounds were identified by comparing their retention times and UV–Vis and mass spectra with standards, when available. Otherwise, compounds were identified tentatively by comparing data obtained from available information published in the literature. For quantitative analysis, calibration curves obtained from available standards or closely related standards were used. The results were expressed as mg/g of extract.

### 2.4. Evaluation of the Bioactive Properties of the Extract 

#### 2.4.1. Antioxidant Activity

The antioxidant activity was evaluated by the DPPH radical scavenging, ferric reducing power (RP), oxidative hemolysis inhibition (OxHLIA), and thiobarbituric acid reactive substances (TBARS) assays. 

The DPPH assay was performed using the DPPH radical (6 × 10^−5^ mol/L), and the radical scavenging activity (RSA) was calculated using the equation

%RSA = [(ADPPH − AS)/ADPPH] × 100 (1)

where AS is the absorbance of the sample solution, and ADPPH is the absorbance of the DPPH solution. 

The ferric reducing power assay was performed using potassium ferricyanide (1% *w/v*, 0.5 mL) and ferric chloride (0.1% *w/v*, 0.16 mL), and the absorbance was measured at 690 nm on a microplate reader (Bio-Tek Instruments, ELX800).

%RP = [(AB − AS)/AB] × 100 (2)

where AS is the absorbance of the sample solution, and AC is the absorbance of the blank (extraction solvent).

The oxidative hemolysis inhibition assay (OxHLIA) was performed in AAPH (160 mM in PBS)-induced erythrocyte solution (2.8%, *v/v*; 200 µL). The optical density was measured at 690 nm on a microplate reader (Bio-Tek Instruments, ELX800) over time until complete hemolysis [23]. The percentage of the erythrocyte population that remained intact (P) was calculated according to the equation

P (%) = (S_t_ − CH_0_/S_0_ − CH_0_) × 100 (3)

where S_t_ and S_0_ correspond to the optical density of the sample at *t* and 0 min, respectively, and CH_0_ is the optical density of the complete hemolysis at 0 min. 

The TBARS assay was performed in brain porcine tissue in the presence of FeSO_4_ (10 mM; 0.1 mL), ascorbic acid (0.1 mM; 0.1 mL), trichloroacetic acid (28%, *w/v*, 0.5 mL), and thiobarbituric acid (TBA, 2%, *w/v*, 0.38 mL). Lipid peroxidation inhibition (%) was calculated using the following expression:

[(A − B)/A] × 100%(4)

where A and B are the absorbance of the control and the sample solution, respectively. For all the antioxidant assays, the results are presented as the extract concentration that provided 50% inhibition (EC_50_) and were calculated from the graph of percentage inhibition against extract concentration.

#### 2.4.2. Anti-Tyrosinase activity

The tyrosinase inhibitory activity of the extracts was determined as previously described [24]. Each extract was dissolved in 50% (*v/v*) ethanol and submitted to successive dilutions. Kojic acid and 3,4-dihydroxy-L-phenylalanine (L-DOPA) were utilized as the positive control and substrate, respectively. Briefly, 1 mL of 2.5 mM L-DOPA solution was mixed with 1.8 mL of 0.1 M phosphate buffer (pH 6.8), followed by the addition of 0.1 mL of the sample solution. The mixture was incubated at 30 °C for 10 min, followed by the addition of 0.1 mL of the tyrosinase enzyme solution (142 Units/mL). The absorbance was measured at 475 nm using a UV–visible spectrophotometer (UV-1600 PC, VWR, Pennsylvania, United States). Tyrosinase inhibition was calculated using the expression [(ABlank − ASample)/ABlank] × 100, and the EC_50_ values were calculated using the calibration curve of tyrosinase inhibition percentage versus extract concentration.

#### 2.4.3. Anti-Inflammatory Activity

The anti-inflammatory activity of the extracts was evaluated in a lipopolysaccharide (LPS)-stimulated murine macrophage (RAW 264.7) cell line by determining the levels of nitric oxide (NO) production. For each experiment, the cells were seeded in 96-well plates at 150,000 cells/well and allowed to attach to the plate overnight. Then, cells were treated with the different concentrations of the extract, and dexamethasone was used as the positive control for 1 h. NO production was quantified using the Griess Reagent System kit containing sulphanilamide, *N*-1-naphthylethylenediamine dihydrochloride (NED), and nitrite solutions, following a procedure previously described [25]. The absorbance was measured at 540 nm (microplate reader ELX800 Biotek) to determine the amount of NO produced.

#### 2.4.4. Antibacterial Activity against Skin Pathogenic Bacteria

The antibacterial activity of the extracts was tested against strains isolated from skin: *Staphylococcus lugdunensis*, *Proteus vulgaris*, and *Staphylococcus epidermidis*, and the reference strain *Staphylococcus aureus* ATCC 6538, by the microdilution method in 96-well microtiter plates. The minimum inhibitory concentrations (MICs) and minimal bactericidal concentrations (MBCs) of the extracts were determined. Streptomycin was used as the positive control.

#### 2.4.5. Cytotoxicity

The cytotoxicity of the extracts was tested on a spontaneously immortalized human keratinocyte cell line (HaCaT) using the crystal violet assay as described previously [26] with some modifications. The extracts were dissolved in phosphate-buffered saline (PBS) to a final concentration of 8 mg/mL. HaCaT cells were grown in high-glucose Dulbecco’s Modified Eagle Medium (DMEM) supplemented with 10% fetal bovine serum (FBS), 2 mM L-glutamine, and 1% antibiotic-antimycotic (Invitrogen), at 37 °C in 5% CO_2_. Forty-eight h before treatment, cells (10^4^) were seeded per well in a 96-well microtiter plate with an adhesive bottom. After 48 h, the medium was removed, and fresh medium with various concentrations of the extracts was added to the cells. The cells were treated with the extracts for 24 h. Afterward, the medium was removed, and the cells were washed twice with PBS and stained with 0.4% crystal violet staining solution for 20 min at room temperature. Then, the crystal violet staining solution was removed, and the cells were washed in a stream of tap water and left to air dry at room temperature. The absorbance of the dye dissolved in methanol was measured at 570 nm (OD_570_) on a plate reader. The results were expressed as the relative growth rate (%) of HaCaT-treated cells compared to untreated control HaCaT cells. Experiments were performed in triplicate for each concentration of the extracts, and three independent experiments were performed. The solvent was used as a negative control. 

#### 2.4.6. Wound Scratch Healing

The assay was performed as described in Stojković et al. [27] with some modifications. HaCaT cells were grown until reaching confluence. The cell monolayer was scratched with a 200 μL tip. Floating cells were washed and incubated in DMEM supplemented with 1% FBS, 2 mM L-glutamine, and 1% antibiotic-antimycotic (Invitrogen), containing 200 µg/mL of extracts. Cell migration was monitored with a Nikon Eclipse TS2 microscope (Amsterdam, The Netherland) 24 h after the wound was created. Untreated cells were used as the control. The results were presented as percentages of wound closure during the exposure to the extracts. Three independent experiments were performed.

#### 2.4.7. Cell Invasion

The assessment of the extracts’ ability to reduce invasion capacities against the bacteria with the lowest MIC towards HaCaT cells was determined as previously described by Ahmed et al. [28] with slight modifications. Briefly, HaCaT cells were grown in 24-well plates with an adhesive bottom until confluence. Then, the medium was removed, and fresh DMEM without FBS containing extracts at sub-inhibitory concentrations of 200 µg/mL was added to the cells. After 15 min incubation at 37 °C, 100 µL of bacterial culture (10^8^ CFU/mL) was added to the cells and incubated for 2 h at 37 °C. Afterward, the medium was removed, and cells were treated with gentamicin (300 µg/mL) for 1 h to kill the adherent bacterial cells. Cells were washed three times with DMEM without FBS and lysed for 30 min at 37 °C with 1 mL of 1% (*v/v*) Tween-20 (Sigma Aldrich, Mannheim, Germany). Subsequently, dilutions of the bacterial suspension in each well were produced and seeded on Trypton Soy Agar plates. After 18 h incubation at 37 °C, the number of CFUs was determined. 

#### 2.4.8. Photostability

Extracts obtained from *J. regia* leaves were dissolved in buffer solutions containing citric acid and sodium citrate (1–3 mg/mL and pH 3–6). The studied pH range is within a healthy skin pH, and it is acceptable for topical application [29]. Plates containing the extracts were irradiated for 90 min using a UV irradiation chamber (UV-consulting Peschl, Castellón, Spain) delivering a flux of 45 mJ/cm^2^ of UVA and UVB radiation. Afterwards, samples were analyzed for total phenol content using the Folin–Ciocalteu reagent, and the antioxidant activity was measured by the DPPH method [2]. 

### 2.5. Formulation Development

#### 2.5.1. Incorporation of the Extract

The cosmetic cream used in this study was the Versatile^™^ enriched oil in water (O/W) cream. This vehicle contains natural lipids that give Versatile^™^ Rich its moisturizing and protective properties, and it allows the development of enriched formulations as it remains consistent with a wide range of active ingredients. It was purchased from Fagron Iberica S.A.U. (Barcelona, Spain). According to the supplier, this product is free from parabens, boric acid, formaldehyde donors, mineral oils/Vaseline, propylene glycol, benzyl alcohol or benzyl benzoate, and peanut oil (https://gr.fagron.com/en-gr/product-innovations/versatiletm accessed on 29 March 2022). According to the supplied technical data, this base cream is produced under GMP regulations with pharmaceutically tested ingredients. The formulations were prepared considering the overall bioactivity results. Hence, 30 mg of extract was incorporated in 1 g of base cream. The formulation was carefully mixed to guarantee sample homogeneity and stored in a light-protective and airtight container at different storage conditions (4, 20, and 40 °C). For the sake of simplicity, the formulations with hydroethanolic extracts stored at 5, 20, and 40 °C were labeled H5, H20, and H40, respectively, while the controls (base cream alone) stored at 5, 20, and 40 °C were labeled C5, C20, and C40, respectively. The antioxidant activity, phenolic profile, color, and pH of the formulations were evaluated over time (0–30 days), using the methodologies described in this section. 

#### 2.5.2. Antioxidant Activity and Phenolic Profile

To perform these assays, 1 g of each formulation was extracted with methanol (20 mL) for 60 min. The sample was centrifuged twice, and the supernatant was collected for bioactivity evaluation and chemical characterization of the phenolic profile [6]. The antioxidant activity was determined by the DPPH radical scavenging assay as previously described. The samples were filtered using a 0.22 µm nylon syringe filter and analyzed by HPLC-DAD-ESI/MS^n^ to identify the individual phenolic compounds present in the formulation [22]. 

#### 2.5.3. Color and pH Measurements

The color of the formulations was measured using a colorimeter (Konica Minolta Sensing Inc., Tokyo, Japan). The L*a*b* color system was used, where L* is a measure of the lightness, a* is a measure of the greenness/redness, and b* is a measure of the blueness/yellowness. The pH of the formulation was determined by mixing 1 g of formulation with 9 mL of distilled water. The pH of the resulting mixture was measured with a pH meter calibrated before each measurement (Hanna Instruments, Woonsocket, RI, USA).

### 2.6. Statistical Analysis

All assays were performed in triplicate, and the results were expressed as the mean ± standard deviation (SD). Statistical analysis of the data was performed using SPSS software (Version 25; IBM Corp., New York, NY, USA). One-way ANOVA was used to determine the differences between test samples (*p* < 0.05). The post hoc comparison was performed using Tukey’s HSD test. 

## 3. Results and Discussion 

### 3.1. Composition in Phenolic Compounds

**Figure 1:**
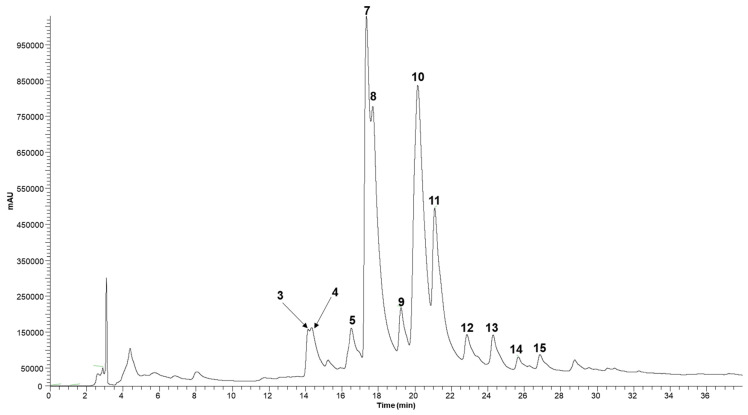
HPLC profile of *J. regia* hydroethanolic extract at 370 nm wavelength. The peak identification and quantification are presented in Table 1.

Fifteen compounds were detected (Table 1, Figure 1), and two of them were classified as phenolic acids identified according to their UV spectra and deprotonated ions. 

Peak 1 ([M-H]^-^ at *m/z* 353) was identified as 3-*O*-caffeoylquinic acid, while peak 2 ([M-H]^-^ at *m/z* 337) was identified according to its MS^2^ fragmentation as an isomer of *p*-coumaroylquinic acid. The identification was assigned based on the patterns reported for the caffeoylquinic acid isomers as previously reported [30,31]. Peaks 3, 4, and 7 were identified as taxifolin derivatives according to their UV spectra and deprotonated ion [M-H]^-^ at *m/z* 435, releasing a fragment at *m/z* 303 [taxifolin-H]^−^ (−132 mu, loss of a pentosyl moiety). ESI/MS analysis does not provide information about the nature and position of the sugar moieties. The other phenolic compounds corresponded to flavonol derivatives derived from quercetin (λmax around 350 nm, MS^2^ fragment at *m/z* 301), kaempferol (λmax around 330 nm, MS^2^ fragment at *m/z* 285), and myricetin (λmax around 340 nm, MS^2^ fragment at *m/z* 317) (Table 1). Quercetin 3-*O*-glucoside (peak 8) was positively identified according to its retention, mass, and UV–Vis characteristics by comparison with commercial standards. Peaks 8 and 9 ([M-H]^-^ at *m/z* 463), and 10, 11, and 12 ([M-H]^-^ at *m/z* 433, 433, and 447, respectively) were assigned to quercetin derivatives; peaks 13 and 14 ([M-H]^-^ at *m/z* 417), and 15 ([M-H]^-^ at *m/z* 431) were assigned to kaempferol derivatives; and peaks 5 and 6 ([M-H]^-^ at *m/z* 449 and 463, respectively) were assigned to myricetin derivatives. 

Moreover, according to the results of the quantitative analysis, the most abundant phenolic compounds in the hydroethanolic extract of *J. regia* leaves were 3-*O*-caffeoylquinic acid (18.30 ± 0.04 mg/g extract), quercetin-*O*-pentoside (9.64 ± 0.06 mg/g extract), a taxifolin *O*-pentoside isomer (9.10 ± 0.04 mg/g extract), quercetin 3-*O*-glucoside (6.70 ± 0.19 mg/g extract), and 3-*p*-coumaroylquinic acid (5.78 ± 0.30 mg/g extract). Santos et al. [32] detected twenty-five compounds in the decoction and methanolic extracts of *J. regia* leaves obtained from the north-eastern Portuguese region of Trás-os-Montes. Five were identified as phenolic acid derivatives (hydroxycinnamic acid derivatives), while the remaining phenolic compounds corresponded to flavonoids and their derivatives. 3-*O*-Caffeoylquinic acid and quercetin *O*-pentoside were the most abundant phenolic compounds in both the decoction and methanolic extracts, and this is similar to what was obtained in this work. Similarly, Zhao et al. [11] reported the presence of eighteen phenolic compounds in a *J. regia* hydroethanolic extract obtained from Tianjin, China. The authors reported the presence of only two phenolic acids (caffeoylquinic acid and 3-*p*-coumaroylquinic acid), which is similar to what is reported in the present work. Even though the extraction methods utilized by the authors were different (microwave extraction), a similar phenolic composition was observed. Other hydroethanolic extracts obtained by heat-assisted extraction, using the same optimized conditions for the extraction of phenolic compounds, were previously reported by [21,33]. In the former work, the three responses used in the optimization study were the three major compounds: 3-*O*-caffeoylquinic acid, quercetin 3-*O*-glucoside, and quercetin-*O*-pentoside, similar to our results. In the second work, two samples collected in Bragança, Portugal, at different phenological stages (green and yellow leaves) presented six phenolic acids, with 3-*O*-caffeoylquinic acid (3.5 ± 0.4 mg/g of extract) and trans-3-*p*-coumaroylquinic acid (6.9 ± 0.5 mg/g of extract) being the most abundant in the green leaves. 

These results demonstrate that hydroxycinnamic acid derivatives such as 3-*O*-caffeoylquinic acid and 3-*p*-coumaroylquinic acid are very common phenolic acids found in *J. regia* leaves, regardless of the cultivar, maturity stage, or method of extraction. In addition, *J. regia* extract also proved to be a source of structurally diverse compounds belonging to the flavonoid class, including flavanols (myricetin, quercetin, and kaempferol derivatives) and flavanonols (taxifolin derivatives). Hence, *J. regia* leaves can be an important source of these classes of compounds. In addition, compounds such as epicatechin, laricitrin, naringenin, isorhamnetin, and procyanidin derivatives have been identified in some *J. regia* leaf extracts [11,32,34]. 

### 3.2. Bioactive Properties of J. regia Extracts

#### 3.2.1. Antioxidant Activity

The antioxidant potential of the studied extracts was assessed by four different in vitro assays, and the results are presented in Table 2. 

The EC_50_ values of the first assays follow the order DPPH > RP > TBARS, varying between 11.8 and 137 µg/mL. By comparing these results with the EC_50_ value of Trolox (42 μg/mL), a known potent antioxidant used here as a positive control, the extract presented a higher effect in the ferric reducing power assay, with EC_50_ = 27.6 μg/mL. In the other antioxidant assays, though the results show a lower antioxidant effect than Trolox, relevant DPPH radical scavenging and lipid peroxidation inhibition activity was displayed.

Comparatively, methanolic extracts obtained from these Portuguese cultivars of *J. regia* also showed interesting antioxidant capacities, with EC_50_ values of 65.91 ± 6.47, 75.87 ± 2.41, and 20.36 ± 0.82 µg of extracts/mL for the DPPH, reducing power, and TBARS assays, respectively [32]. These authors reported a different antioxidant capacity trend in EC_50_ values (RP > DPPH > TBARS).

Regarding the OxHLIA assay, the results also show the potential of the studied extract to inhibit the oxidative hemolysis of erythrocyte cells from the hemolytic action caused by the 2,2′-azobis(2-methylpropionamidine) dihydrochloride-derived peroxyl radical for 60 and 120 min. At 60 min, the *J. regia* hydroethanolic extract presented better protection of the erythrocyte population. The protective effect of the hydroethanolic extract after 120 min was comparable to Trolox (43.5 ± 0.3 μg/mL). To the best of our knowledge, only Vieira et al. [33] previously studied the antihemolytic potential of the green and yellow leaves of *J. regia* collected from Bragança, with 32 ± 2 μg/mL and 51 ± 2 μg/mL being the EC_50_ values at Δt ≈ 60 min, respectively. According to the available literature, the bioactive potential of *J. regia* extracts is highly dependent not only on the source of plant material, but also on the extraction conditions [16]. The above authors carried out an extensive work that compared the performance of different extraction solvents, including glycerol and alkanediols, to extract phenolic compounds from *J. regia* leaves. 

#### 3.2.2. Anti-Tyrosinase Activity

Plants are important sources of anti-tyrosinase components such as polysaccharides, phenolic compounds, terpenes, and fatty acids [35]. In the present work, the anti-tyrosinase capacity of the *J. regia* hydroethanolic extract was measured by determining tyrosinase inhibition using L-DOPA as a substrate. Tyrosinase is the rate-limiting enzyme that catalyzes two major steps: L-tyrosine’s hydroxylation to 3,4-dihydroxyphenylalanine (L-DOPA), and the oxidation of L-DOPA to dopaquinone, leading to the formation of melanin [5]. Besides its contribution to skin pigmentation, this enzyme also plays a vital role in neurodegeneration associated with Parkinson’s disease and the browning of vegetables. Hence, extracts with potent tyrosinase inhibition activity can be potential candidates to be incorporated in topical formulations to suppress hyperpigmentation, and as medicinal and anti-browning ingredients [36]. As shown in Table 2, the hydroethanolic extract of *J. regia* presented up to 50% inhibition of tyrosinase at 751 ± 0.01 µg of extract/mL. Previous findings on the tyrosinase inhibitory activity of *J. regia* leaves obtained from Turkey were reported by Uysal et al. [37], but the authors reported very low tyrosinase activity for both the water and methanol extracts (3–6% inhibition) at the highest tested concentration. As far as our knowledge is concerned, this is the first time that a positive anti-tyrosinase activity is reported for *J. regia* extract. Several environmental factors, including climate and altitude, cultivation techniques, and the extraction process employed, may be attributed to the observed differences in the anti-tyrosinase activity [38]. 

#### 3.2.3. Anti-Inflammatory Activity

The anti-inflammatory potential of *J. regia* extract was assessed using RAW 264.7 macrophages cells, and the results are presented in Table 2. An extract concentration of 109 ± 5 μg/mL inhibited half of the NO produced by the macrophage cells, representing the EC_50_ value. The result shows an 18-fold lower effect in comparison to dexamethasone, a corticosteroid drug used in the treatment of many inflammatory conditions. Vieira et al. [34] also reported an EC_50_ of 319 µg/mL for the hydroethanolic extract of the green leaves of *J. regia*, but no activity was found in the yellow leaves. Other studies have shown that extracts obtained from the different parts of *J. regia* such as its ethyl acetate kernel extract [39], aqueous seed extract [40], and hydroethanolic husk extract [14] also presented anti-inflammatory effects in different in vitro and in vivo models. Moreover, only Muzaffer et al. [41] reported that the anti-inflammatory effect of a male flower extract of *J. regia* was associated with significant downregulation of mRNA expression of tumor necrosis factor (TNF-α), interleukins (IL-1 and IL-6), nuclear factor kappa-light-chain-enhancer of activated B cells (NF-κB), and cyclooxygenase enzyme (COX-2). 

#### 3.2.4. Antibacterial Activity

The antibacterial activity of the *J. regia* hydroethanolic extract was assessed against common skin pathogens, considering its potential application in cosmeceutical products. The results are presented in Table 2 as the minimum inhibitory concentration (MIC) and minimum bactericidal concentration (MBC). *J. regia* extract showed the highest antibacterial activity against *P. vulgaris*, with the corresponding lowest MIC and MBC (values with a similar order of magnitude to the positive control). The extract was also effective against *S. lugdunensis* and *S. aureus*, with MICs in the concentration range of 2–4 (mg/mL), while *S. epidermidis* was the least inhibited microbial strain. The results show a better bactericidal effect of the extract against Gram-negative bacterial strains (*P. vulgaris* and *S. lugdunensis*) in comparison to the Gram-positive strains (*S. epidermidis* and *S. aureus*). The increased antimicrobial resistance among Gram-positive bacteria may be associated with the peptidoglycan walls, which act as a protective barrier, that prevent bioactives from penetrating into the cytoplasm [42]. The hydroethanolic extract obtained from *J. regia* yellow and green leaves also showed a stronger antimicrobial effect against Gram-positive bacterial strains such as *Enterococcus faecalis*, *Listeria monocytogenes*, and Methicillin-resistant *Staphylococcus aureus* [33]. However, the authors utilized bacterial strains that were obtained from clinical specimens, and these strains have been shown to be more resistant to potential antimicrobial compounds [43]. To the best of the authors’ knowledge, the antimicrobial potential of *J. regia* leaf extracts against skin pathogens associated with acne lesions was first reported by Qa’dan et al. [44]. The acetone extract was reported to inhibit *Propionibacterium acnes*, *S. aureus*, and *S. epidermidis*. *J. regia* leaf extracts have also shown very promising antimicrobial potential against a broad spectrum of microbial pathogens including *Streptococcus mutans*, *Streptococcus salivarius*, *Streptococcus sanguinis*, *Actinomyces viscosus*, *Bacillus cereus*, *Escherichia coli*, *Pseudomonas aeruginosa*, *Aspergillus niger*, and *Candida albicans* [45,46]. Hence, these findings show that *J. regia* leaf extracts could be utilized as a promising cosmetic additive due to their reported antimicrobial activity against some of the above-mentioned microbial strains that represent common pathogens that colonize the skin and wound infections. 

#### 3.2.5. Cytotoxicity 

**Figure 2:**
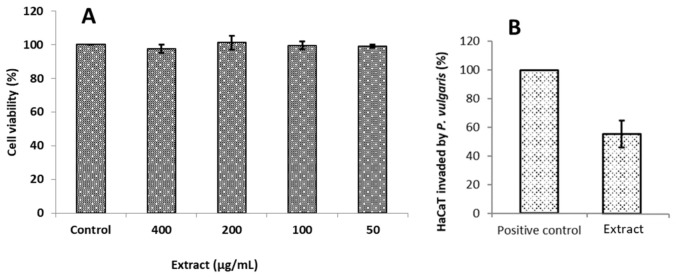
(**A**) Relative growth rate (%) of HaCaT cells treated with different concentrations of *J. regia* extract. (**B**) Relative *P. vulgaris* invasion capacity of HaCaT cells treated with extract (200 µg/mL) presented as percentage of *P. vulgaris* invasion capacity of untreated HaCaT cells (arbitrarily set at 100%).

The effect of the extract concentration on the cell viability of a keratinocyte cell line (HaCaT) was conducted using the crystal violet assay. The results show that at the highest tested concentration, up to 97% viability of HaCaT cells was maintained (Figure 2A). 

Subtoxic concentrations of the extract, which represent the extract concentration that does not reduce the viability of HaCaT cells (200 µg/mL), were further selected to perform the wound healing and the cell invasion assay described in Section 3.2.6 and Section 3.2.7, respectively. To the best of our knowledge, this is the first time this type of cytotoxicity study using skin cell lines has been performed with the extract of *J. regia* leaves. 

Nevertheless, previous studies on the male flower of *J. regia* using a methanolic extract at 110 μg/mL [19] and 80 μg/mL [41] concentrations reported that the cell viability (100%) of UVB-treated HaCaT cells was maintained.

#### 3.2.6. Wound Scratch Healing

The wound healing process is a complex cascade of events in different tissues and cells associated with inflammation, cell proliferation, neovascularization, collagen deposition, epithelialization, and wound contraction [47]. Bioactive ingredients are often used in ethnomedicine in the form of nutraceuticals, oils, or ointments, with anti-inflammatory, antioxidant, and antimicrobial activity and less toxicity effect to treat or accelerate wound healing. Twenty-four hours after the artificial wound was generated, the extract was found to have facilitated 27.86% (±3.68%) wound closure, similar to the result obtained in the control assay using untreated cells (26.24% ± 2.44%), which is an indication that treatment with the extract maintained the migration rate of keratinocyte cells. 

#### 3.2.7. Cell Invasion

Despite a wide variety of defense mechanisms in cultured cells or tissues, bacterial pathogens have the capacity to invade and colonize the surface of host cells and tissues. In the present work, the cell invasion activity was assessed in HaCaT cells, using the subtoxic concentration and the minimum inhibitory concentration of the extract. Hence, the ability to reduce the invasion of *P. vulgaris* towards HaCaT cells was assessed at 200 µg/mL, and the result is presented in Figure 2B. The result shows that the extract presented up to 40% inhibition of *P. vulgaris* invasion compared to untreated HaCaT cells. The present work evaluated the potential of the *J. regia* hydroethanolic extract against *P. vulgaris* within infected HaCaT cells and its ability to promote wound closure in an artificially generated wound. This promising function makes the studied extract a potential candidate in developing novel topical formulations to combat skin infections.

#### 3.2.8. Photostability

*J. regia* hydroethanolic extracts are a rich source of phenolic compounds, including phenolic acids and flavonoids. Due to the multifunctional biological properties of these compounds, there is a renewed interest in their utilization as promising cosmetic sunscreen ingredients due to their potential to absorb a broad spectrum of UV radiation, including the UVB and UVA regions [48]. Despite their sun protection capacities, some of these compounds have been demonstrated to be photo-unstable after long-term exposure to UV radiation. Hence, in the present work, the hydroethanolic extract was subjected to both UVA and UVB, at different pH conditions (pH 3–6). The photostability of the extracts was evaluated according to their DPPH radical scavenging activity and total phenolic content in comparison to samples placed in the dark. 

**Figure 3:**
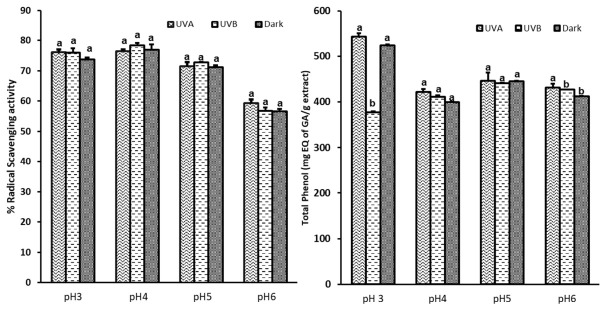
The photostability profile of *J. regia* hydroethanolic extract. Each value is the mean of three replicate determinations ± standard deviation. Means with different letters are significantly different (*p* < 0.05).

As shown in Figure 3, there was no significant difference in the radical scavenging activity of the extract under UVA and UVB conditions, which suggests that UV radiation at the selected intensity did not degrade the activity of the extract, thereby confirming the photostable profile of the studied extract at the different pH conditions. 

By analyzing the total phenol content (TPC) of the UV-exposed extracts, in Figure 3, it is possible to see that the samples at pH = 6, exposed to UVB, and those placed in the dark exhibited very small statistically significant changes in the TPC (less than 3%) in comparison with the UVA-exposed samples. In addition, a significant change in the TPC of the UVB extract at pH = 3 was also observed. Overall, regardless of the intensive UVA or UVB irradiation, the extracts exhibited excellent photostability, especially at pH 4, 5, and 6. The human skin pH values have been reported to range from pH 4.0 to 7.0 in the literature [49]. The photostability profile of bioactive ingredients is an important consideration in the development and performance of sunscreen formulations. The present findings show that the present photostable extract could be further investigated to determine its sun protection factor (SPF). To the best of our knowledge, this is the first time that the photostability of *J. regia* leaf extract has been evaluated. 

Regarding other parts of the plant, the UV-protective effect of a *J. regia* seed extract against oxidative stress induced by a UVB dose of 35 mJ/cm^2^ in keratinocytes after 5–6 h exposure was evaluated by Przełkora et al. [40]. The *J. regia*-treated cells were reported to maintain their normal and typical morphology after UVB exposure, indicating that the *J. regia* aqueous seed extract has a significant UVB radiation-protective effect. 

### 3.3. Development of the Cosmeceutical Formulation 

**Figure 4:**
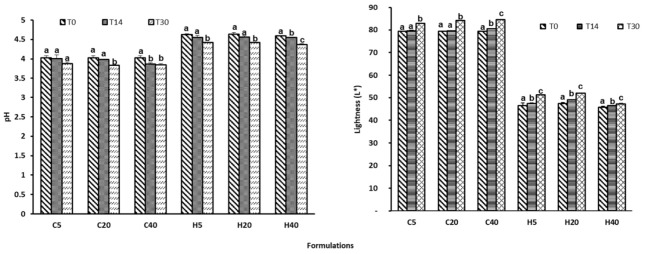
pH and lightness (L*) variation in Formulations 1 and 2 evaluated at different times and temperatures. Each value is the mean of three replicate determinations ± standard deviation. Means with different letters are significantly different (*p* < 0.05).

In the cosmetic industry, extracts and individual compounds obtained from various natural sources are widely used as functional ingredients in a multitude of formulations. However, several studies have reported the physical and chemical instability of these formulations with naturally derived ingredients due to the gradual loss of extract activity over time [50,51]. This has strengthened the need to perform detailed physicochemical characterization and monitor changes in formulation bioactivities [52,53]. For physical stability, the variations in the pH and color of the formulations were tested over time (initial conditions—T0; after 14 days—T14; after 30 days—T30), stored at different temperature conditions (5, 20, and 40 °C). The results obtained for the base cream alone (Formulation 1; samples C5, C20, and C40), used as a control sample, are compared in Table 3 and Figure 4 with the results obtained using the base cream containing the hydroethanolic extract (Formulation 2; samples H5, H20, and H40). As can be seen, by adding the extract, the pH of the formulation increased by only around 0.5–0.7 units. During the 30 days of the assay, the pH values presented minimal changes, varying within the ranges of 4.37–4.63 and 3.83–4.02 for the hydroethanolic formulation and vehicle control, respectively (Figure 4). 

With the exception of the C5 sample, in all cases, only a small statistically significant decrease of 0.2 pH units was measured in both formulations, after 30 days. These results show that the studied formulations were relatively stable between 5 and 40 °C. A similar trend was also reported for hydrogels prepared with a *Castanea sativa* hydroethanolic extract [53]. The above authors reported that gels stored at 4 ºC presented excellent stability, with no significant difference in the pH, color, moisture, and adhesiveness. Color evaluation is a complementary method to assess the acceptance of the product by consumers. The results of the color evaluation are summarized in Table 3 and Figure 4.

**Figure 5:**
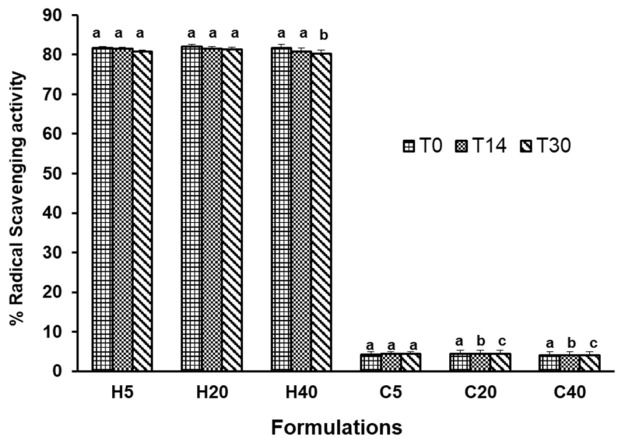
Antioxidant activity of the different formulations at time 0 and after 14 and 30 days of storage at 5, 20, and 40 °C. Each value is the mean of three replicate determinations ± standard deviation. Means with different letters are significantly different (*p* < 0.05).

As can be seen, upon addition of the extract, parameter L* decreased from average values of 80 to 50 when compared to the control sample, maintaining a satisfactory lightness at the different temperatures and times. In both formulations (with or without the extract), after 30 days, a slight increase was observed, with maximum variation of 4.5 in the control formulations and 3.28 in the functionalized creams. These observed differences might be very critical and can be an indication of the significant benefit of the extract to resist changes in the functionalized formulations’ physicochemical properties. In what concerns a*, only the formulations with hydroethanolic extracts exhibited positive values (1.75–3.23), while the control formulation presented negative values. Observing the b* parameter as shown in Table 3, which is an indication of the yellowness index, an increase in yellowness in all the functionalized creams in comparison to the control samples was observed. In relation to the a* parameter, all formulations presented very high positive b* values, which is an indication of the prevalence of yellowness. Bioactive ingredients are usually added to topical formulations to protect the skin from harmful free radicals generated due to UV radiation, thereby preventing the skin from aging, inflammation, and UV-associated skin damage [36,54]. The DPPH assay is one of the most used methods to determine the free radical scavenging activity of extracts, pure compounds, and formulations. In the present work, the radical scavenging activity of the formulations was established to confirm their antioxidant activity over time. The results obtained for both formulations containing the extracts and the base cream are presented in Figure 5.

As aimed, the addition of the extract greatly increased the antioxidant activity of the formulation, from average values of 4% in the control sample to 80% in all the functionalized formulations. Regarding the formulations containing hydroethanolic extracts stored at 5 °C and 20 °C (H5 and H20), an excellent stability profile was observed. There was no statistically significant difference (*p* < 0.05) in the antioxidant activity of these two formulations up to 30 days as shown in Figure 5. Similar to what was observed in the pH analysis, there was only a small statistically significant decrease of 1–2% in the radical scavenging activity of the H40 formulation, after 30 days. The results show that the high antioxidant activity of the final formulation was sustained over time and was not significantly influenced by the temperature of storage and time. The observed sustenance of the antioxidant activity might be associated with the high concentration of bioactive compounds and excellent compatibility with the different formulation ingredients, including emulsifiers, emollient, lubricant, pro-liposome chelating agent, and preservatives. These findings, therefore, fulfil the initial objective of obtaining a final formulation with a beneficial effect and contribute to the scientific evidence that supports the use of *J. regia* leaf extracts as cosmeceutical ingredients. In general, the antioxidant activity of extracts or formulations is correlated with their phenolic compound content [52,53]. Besides phenolic compounds, terpenes and polysaccharides have been identified in cosmetic formulations containing natural extracts, and they have been reported to contribute to their antioxidant and skin hydration properties [55]. In particular, organic acids, tocopherols, and mono- and oligosaccharides have already been identified in different extracts of *J. regia* leaves [32]. In the present work, only phenolic compounds were monitored in the formulations over time and at different storage conditions to ascertain their stability in the vehicle. The results are presented in Table 4. 

All the compounds (3-*O*-caffeoylquinic acid, 3-*p*-coumaroylquinic acid, taxifolin -*O*-pentoside isomer, quercetin 3-*O*-glucoside, quercetin 3-*O*-hexoside, quercetin *O*-pentoside, quercetin *O*-rhamnoside, kaempferol *O*-pentoside, kaempferol *O*-rhamnoside) were successfully identified based on their retention times and UV–Vis and mass spectra, and the chromatogram of each formulation is presented in Figure S1. In the formulation stored at 5 °C (H5), the amounts of peak 2, 3, 10, 11, and 12, identified as 3-*p*-coumaroylquinic acid, taxifolin *O*-pentoside isomer, kaempferol *O*-pentoside, kaempferol *O*-pentoside, and kaempferol *O*-rhamnoside, respectively, were not statistically different up to 14 days. In the H20 formulation, only quercetin 3-*O*-glucoside, kaempferol *O*-pentoside, and kaempferol *O*-pentoside were found to not differ statistically up to 14 days, while in the H40 formulation, only kaempferol *O*-rhamnoside preserved its stability. In comparison to the other compounds as shown in Table 4 a statistically significant decrease in their amount was observed in all three studied formulations over time. This is an indication that the time and temperature of storage contributed significantly to the gradual degradation of the identified bioactive compounds present in the formulations.

Samples stored at lower temperatures usually slow down physicochemical changes that may occur in cosmetic formulations. However, temperatures between 40 and 45 °C are often recommended as maximum testing temperatures to ascertain the performance of formulations in more adverse conditions that are less likely to occur. Overall, the formulations stored at 40 °C presented significant changes in their phenolic content over time. In comparison with the effect observed in the radical scavenging activity, the results show that the antioxidant activity of the formulations was not statistically different, as shown in Figure 3. This is also an indication of the presence of other bioactive metabolites not quantified in the present work that are also important contributors to the antioxidant effect of the studied formulations. Antioxidant extracts and individual compounds, especially those rich in phenolic compounds, have been widely utilized as functional ingredients in cosmetic formulas. However, long-term studies on the antioxidant activity of these formulations have been disappointing, and it appears some of these naturally occurring bioactive compounds present poor stability [6,52]. This degradation or loss of bioactive compounds might influence the color, pH, and viscosity of the formula, thereby contributing to its ineffectiveness and instability. Hence, more work in the context of stability and shelf life still needs to be conducted involving specific stability strategies to evaluate the impact of longer storage conditions on the functional and bioactive properties of these formulations.

## 4. Conclusions

The utilization of natural extracts in cosmetic formulations as antiaging, anti-inflammatory, or photoprotection products has high potential in the current market. This is associated with the increased awareness among consumers of the origin, safety, and environmental fate of cosmetic additives. The possibility of upscaling these by-products from agro-industrial processes contributes to cosmetic sustainability and economic return with innovative products. *Juglans regia* by-products (husk, leaves, and kernels) have been widely used in folk medicine to treat various diseases. Recently, increased attention has been placed on the bioactive composition and biological properties of extracts obtained from *J. regia* by-products. Moreover, the potential of *J. regia* extracts and their bioactive molecules remains only partially explored for cosmeceutical application. The present study shows that a *J. regia* hydroethanolic extract was rich in biologically active metabolites, including phenolic acids and flavonoids. The prepared extract also presented relevant antioxidant, anti-tyrosinase, anti-inflammatory, photostable, and antibacterial effects against skin pathogens. The displayed biological properties and lack of toxicity in the skin cell line make the studied extract potentially suitable for direct inclusion into cosmeceutical formulations for topical application. Moreover, our result also indicates that after incorporating the extract into a cosmetic base cream, the final formulation presented an excellent stability profile over time (30 days) and at different storage conditions, with additional functionality (antioxidant activity was maintained). Even though progress has been made concerning the use of *J. regia* extracts as cosmeceutical ingredients, their safety and efficacy lack the appropriate reporting and dissemination. Furthermore, the safety evaluation of ingredients for cosmeceutical applications is a critical step as some of these natural extracts contain different biomolecules that may be irritants to skin cells. Hence, studies involving skin irritation, eye irritation, genotoxicity, photo-irritation, and dermal absorption should be performed to further strengthen the cosmeceutical potential of *J. regia* leaves. The stability of the extract in the formulation should also be assessed over longer periods of time.

## Figures and Tables

**Table 1:** Identification and quantification of phenolic compounds in *J. regia* hydroethanolic extract.

Peak	Rt (min)	λmax (nm)	[M-H]¯(*m/z*)	MS^2^ (*m/z*)	Tentative Identification	Quantification (mg/g Extract)
1	4.4	324	353	191 (100), 179 (50), 173 (19), 135 (10)	3-*O*-Caffeoylquinic acid ^1^	18.30 ± 0.04
2	5.58	311	337	191 (100), 173 (35), 163 (100)	3-*p*-Coumaroylquinic acid ^2^	5.78 ± 0.30
3	14.15	350	435	303 (20), 285 (100)	Taxifolin *O*-pentoside isomer ^3^	0.47 ± 0.01
4	14.34	349	435	303 (41), 285 (100)	Taxifolin *O*-pentoside isomer ^3^	1.06 ± 0.02
5	16.53	343	449	317 (100)	Myricetin *O*-pentoside ^3^	1.51 ± 0.0008
6	16.83	281	463	317 (100)	Myricetin *O*-rhamnoside ^3^	1.91 ± 0.09
7	17.36	354	435	303 (100), 285 (31)	Taxifolin *O*-pentoside isomer ^3^	5.31 ± 0.02
8	17.7	354	463	301 (100)	Quercetin 3-*O*-glucoside ^3^	6.70 ± 0.19
9	19.25	351	463	301 (100)	Quercetin 3-*O*-hexoside ^3^	2.01 ± 0.0039
10	20.17	352	433	301 (100)	Quercetin *O*-pentoside ^3^	9.64 ± 0.06
11	21.1	348	433	301 (100)	Quercetin *O*-pentoside ^3^	5.12 ± 0.16
12	22.86	342	447	301 (100)	Quercetin *O*-rhamnoside ^3^	1.62 ± 0.01
13	24.29	329	417	285 (100)	Kaempferol *O*-pentoside ^3^	1.29 ± 0.0018
14	25.68	332	417	285 (100)	Kaempferol *O*-pentoside ^3^	0.79 ± 0.0023
15	26.87	334	431	285 (100)	Kaempferol *O*-rhamnoside ^3^	0.90 ± 0.0026
					Total phenolic acids	24.08 ± 0.35
					Total flavonoids	38.33 ± 0.59
					Total phenolic compounds	62.41 ± 0.94

Calibration curves used: ^1^ chlorogenic acid, y = 168,823 x − 161,172, *r^2^* = 0.999; ^2^ *p*-coumaric acid, y = 301,950 x + 6966.7, *r^2^* = 0.999; ^3^ quercetin-3-*O*-glucoside, y = 34,843 x − 160,173, *r^2^* = 0.999.

**Table 2:** Bioactive properties of *J. regia* hydroethanolic extract.

Bioactivity	Hydroethanolic Extract	Positive Control
Antioxidant activity (EC_50_)	(µg/mL)	
DPPH scavenging activity	137 ± 10	41 ± 1.1
Reducing power	27.6 ± 0.02	42 ± 0.9
TBARS formation inhibition	11.83 ± 1.06	5.41 ± 0.3
OxHLIA		
Δt = 60 min	10.8 ± 0.5	21.8 ± 0.2
Δt = 120 min	51 ± 1	43.5 ± 0.3
Anti-tyrosinase activity (EC_50_)	µg/mL	µg/mL
L-DOPA inhibition	751 ± 0.01	4.26 ± 0.02
Anti-inflammatory activity (EC_50_)	(µg/mL)	
NO inhibition	109 ± 5	6 ± 0.1
Antibacterial activity (MIC/MBC)	(mg/mL)	
*Staphylococcus lugdunensis*	2/2	0.003/0.006
*Proteus vulgaris*	0.25/0.5	0.1/0.2
*Staphylococcus epidermidis*	4/8	0.003/0.006
*Staphylococcus aureus* (ATCC 6538)	2/4	0.04/0.1

nd—not determined; Trolox, kojic acid, dexamethasone, and streptomycin were used as a positive control for the antioxidant, anti-tyrosinase, anti-inflammatory, and antibacterial activities, respectively.

**Table 3:** Color variation (L*, a*, and b*) of control base cream and formulations containing *J. regia* hydroethanolic extracts.

**Formulation 1 (Base cream)**									
	**C5**			**C20**			**C40**		
	**T0**	**T14**	**T30**	**T0**	**T14**	T30	T0	T14	T30
L*	79.34 ± 0.42	79.6 ± 0.63	82.93 ± 0.21	79.34 ± 0.42	79.67 ± 0.83	84.13 ± 0.47	79.34 ± 0.42	80.56 ± 0.26	84.46 ± 0.25
a*	−1.52 ± 0.01	−1.10 ± 0.03	−1.24 ± 0.01	−1.52 ± 0.01	−1.16 ± 0.01	−1.15 ± 0.01	−1.52 ± 0.01	−1.07 ± 0.01	−1.59 ± 0.01
b*	0.79 ± 0.01	1.05 ± 0.02	1.32 ± 0.07	0.79 ± 0.01	1.12 ± 0.14	1.28 ± 0.01	0.79 ± 0.01	1.17 ± 0.01	0.46 ± 0.05
	**Formulation 2 (Hydroethanolic extract formulation)**								
	**H5**			**H20**			**H40**		
	T0	T14	T30	T0	T14	T30	T0	T14	T30
L*	46.47 ± 0.22	47.39 ± 0.32	51.15 ± 0.06	47.30 ± 0.20	48.44 ± 0.08	51.88 ± 0.09	45.64 ± 0.16	46.39 ± 0.20	47.14 ± 0.10
a*	1.99 ± 0.2	1.75 ± 0.09	2.17 ± 0.01	1.8 ± 0.12	1.65 ± 0.01	2.43 ± 0.01	1.9 ± 0.03	2.43 ± 0.04	3.23 ± 0.03
b*	13.48 ± 0.25	14.03 ± 0.23	20.75 ± 0.08	13.67 ± 0.03	14.083 ± 0.2	20.84 ± 0.15	12.47 ± 0.14	13.87 ± 0.06	19.2 ± 0.17

**Table 4:** The phenolic compound profile of each hydroethanolic formulation at different storage conditions in mg/g of cream.

Peaks	H5 Formulation			H20 Formulation			H40 Formulation		
	T0	T14	T30	T0	T14	T30	T0	T14	T30
1	0.62 ± 0.1 ^a^	0.61 ± 0.09 ^b^	0.49 ± 0.02 ^c^	0.77 ± 0.12 ^a^	0.71 ± 0.12 ^b^	0.49 ± 0.01 ^c^	0.67 ± 0.1 ^a^	0.57 ± 0.1 ^b^	0.48 ± 0.02 ^c^
2	0.76 ± 0.1 ^a^	0.76 ± 0.05 ^a^	0.63 ± 0.01 ^b^	0.86 ± 0.1 ^a^	0.82 ± 0.1 ^b^	0.63 ± 0.03 ^c^	0.80 ± 0.11 ^a^	0.73 ± 0.1 ^b^	0.65 ± 0.01 ^c^
3	0.50 ± 0.05 ^a^	0.51 ± 0.06 ^a^	0.40 ± 0.01 ^b^	0.53 ± 0.07 ^b^	0.56 ± 0.04 ^a^	0.40 ± 0.02 ^c^	0.60 ± 0.08 ^a^	0.50 ± 0.05 ^b^	0.38 ± 0.02 ^c^
4	0.48 ± 0.07 ^a^	0.45 ± 0.01 ^b^	0.36 ± 0.01 ^c^	0.48 ± 0.05 ^a^	0.48 ± 0.08 ^a^	0.35 ± 0.01 ^b^	0.50 ± 0.06 ^a^	0.44 ± 0.04 ^b^	0.36 ± 0.03 ^c^
5	2.72 ± 0.1 ^a^	2.74 ± 0.1 ^b^	1.51 ± 0.02 ^c^	3.12 ± 0.1 ^a^	2.95 ± 0.11 ^b^	1.39 ± 0.02 ^c^	3.10 ± 0.15 ^a^	2.36 ± 0.13 ^b^	1.57 ± 0.01 ^c^
6	0.43 ± 0.02 ^a^	0.40 ± 0.01 ^b^	0.33 ± 0.01 ^c^	0.42 ± 0.03 ^a^	0.45 ± 0.03 ^b^	0.32 ± 0.03 ^c^	0.43 ± 0.01 ^a^	0.39 ± 0.05 ^b^	0.35 ± 0.01 ^c^
7	1.87 ± 0.1 ^a^	1.86 ± 0.1 ^b^	1.16 ± 0.02 ^c^	2.24 ± 0.1 ^a^	2.13 ± 0.1 ^b^	1.06 ± 0.02 ^c^	2.33 ± 0.12 ^a^	1.79 ± 0.05 ^b^	1.18 ± 0.01 ^c^
8	1.37 ± 0.1 ^a^	1.35 ± 0.15 ^b^	0.82 ± 0.01 ^c^	1.57 ± 0.1 ^a^	1.48 ± 0.11 ^b^	0.76 ± 0.01 ^c^	1.41 ± 0.1 ^a^	1.18 ± 0.06 ^b^	0.90 ± 0.02 ^c^
9	0.50 ± 0.04 ^b^	0.54 ± 0.02 ^a^	0.39 ± 0.01 ^c^	0.55 ± 0.05 ^a^	0.51 ± 0.01 ^b^	0.37 ± 0.01 ^c^	0.52 ± 0.05 ^a^	0.47 ± 0.03 ^b^	0.40 ± 0.03 ^c^
10	0.44 ± 0.07 ^a^	0.44 ± 0.03 ^a^	0.35 ± 0.01 ^b^	0.44 ± 0.08 ^a^	0.43 ± 0.05 ^a^	0.34 ± 0.02 ^b^	0.45 ± 0.02 ^a^	0.40 ± 0.02 ^b^	0.34 ± 0.01 ^c^
11	0.35 ± 0.02 ^a^	0.34 ± 0.02 ^a^	0.31 ± 0.02 ^b^	0.36 ± 0.02 ^a^	0.36 ± 0.01 ^a^	0.31 ± 0.03 ^b^	0.36 ± 0.01 ^a^	0.33 ± 0.01 ^b^	0.32 ± 0.02 ^c^
12	0.42 ± 0.03 ^a^	0.42 ± 0.05 ^a^	0.33 ± 0.02 ^b^	0.45 ± 0.02 ^a^	0.38 ± 0.04 ^b^	0.33 ± 0.02 ^c^	0.40 ± 0.02 ^a^	0.38 ± 0.02 ^a^	0.33 ± 0.01 ^b^
TPA	1.39 ± 0.2	1.38 ± 0.14	1.13 ± 0.03	1.64 ± 0.22	1.54 ± 0.22	1.13 ± 0.04	1.47 ± 0.21	1.30 ± 0.2	1.14 ± 0.03
TF	9.13 ± 0.6	9.10 ± 0.55	6.02 ± 0.14	10.18 ± 0.62	9.78 ± 0.58	5.69 ± 0.19	10.16 ± 0.62	8.28 ± 0.46	6.18 ± 0.17
TPC	10.52 ± 0.8	10.14 ± 0.69	7.15 ± 0.17	11.82 ± 0.84	11.32 ± 0.8	6.82 ± 0.23	11.64 ± 0.83	9.59 ± 0.66	7.33 ± 0.2

1: 3-*O*-caffeoylquinic acid; 2: 3-*p*-coumaroylquinic acid; 3: taxifolin *O*-pentoside isomer; 4: quercetin 3-*O*-glucoside; 5: quercetin 3-*O*-glucoside; 6: quercetin 3-*O*-hexoside; 7: quercetin *O*-pentoside; 8: quercetin *O*-pentoside; 9: quercetin *O*-rhamnoside; 10: kaempferol *O*-pentoside; 11: kaempferol *O*-pentoside; 12: kaempferol *O*-rhamnoside; TPA: total phenolic acids; TF: total flavonoids; TPC: total phenolic compounds. Each value represents the mean ± SD. In each row, different letters indicate a significant difference (*p* < 0.05).

## Data Availability

Data are contained within the manuscript and Supplementary Material.

## Supplementary Materials

The following supporting information can be downloaded at: https://www.mdpi.com/article/10.3390/antiox11040677/s1, Figure S1: HPLC profile of each formulation containing *J. regia* hydroethanolic extract at different storage temperature.
