# Supplementary material for: Valorization of Juglans regia Leaves as Cosmeceutical Ingredients: Bioactivity Evaluation and Final Formulation Development
Source: Antioxidants (Basel). 2022 Mar 30;11(4):677. doi: 10.3390/antiox11040677 (PMC9031312; doi:10.3390/antiox11040677)
Supplement: Supplementary file 1 [file antioxidants-11-00677-s001.zip › antioxidants-1635938-supplementary.pdf]

Supplementary material

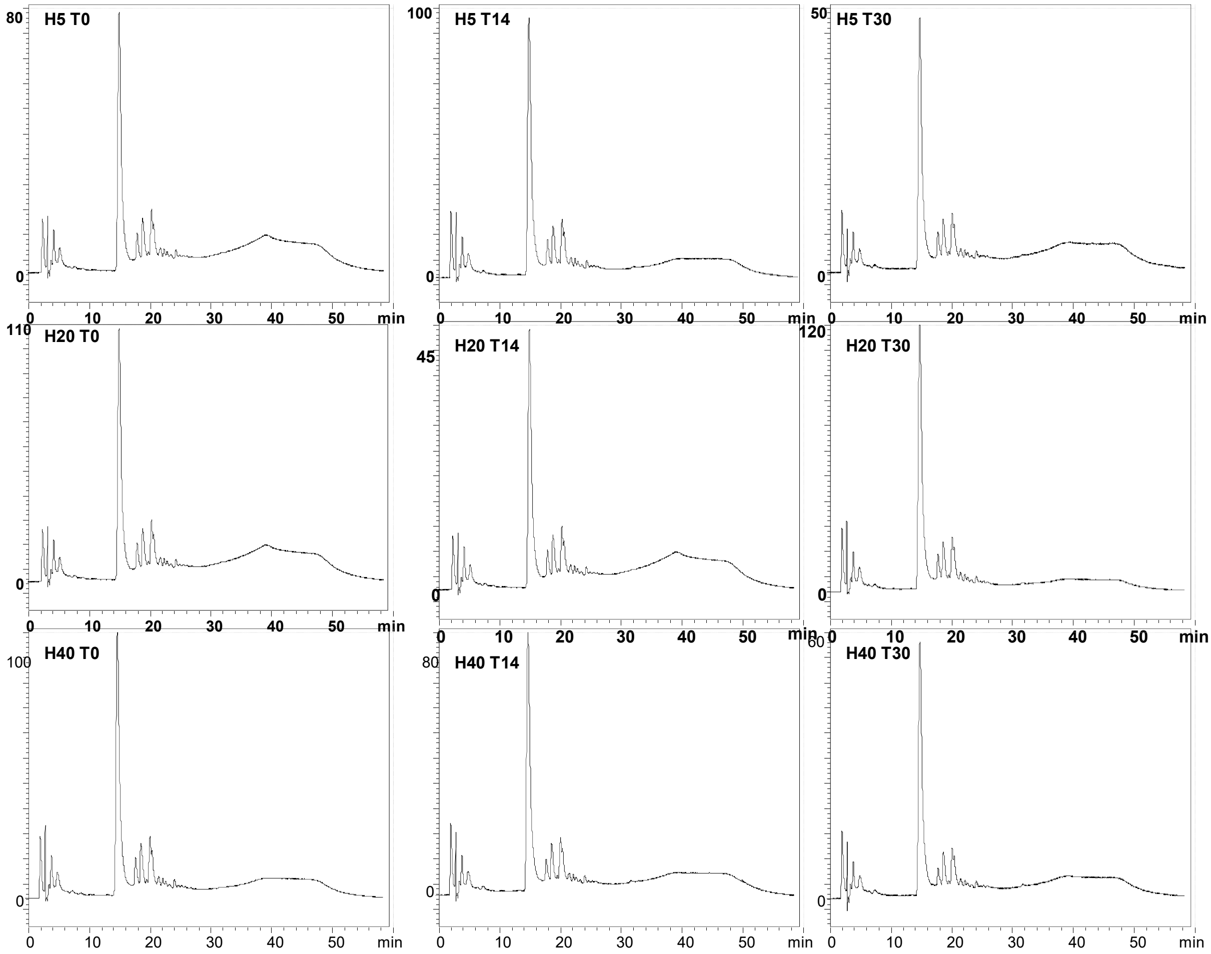

Figure S1. HPLC profile of each formulation containing *J. regia* hydroethanolic extract at different storage temperature.
